# Damp-Heat Ageing of Resin Insulation Materials (Epoxy Resin and Phenolic Resin as Examples) and Their Effects on Flame Retardancy

**DOI:** 10.3390/polym18101200

**Published:** 2026-05-14

**Authors:** Yue Ming, Xinhan Qiao, Haoran Meng, Wentian Zeng, Xiaolei Xia, Feng Yang, Ke Xu, Zhijin Zhang, Chuanhui Huang

**Affiliations:** 1 School of Mechanical & Electrical Engineering, Xuzhou University of Technology, Xuzhou 221018, China; mingyue12267@xzit.edu.cn (Y.M.); 18086799908@189.cn (X.X.); xzit_yf@126.com (F.Y.); 2 School of Electrical Engineering, China University of Mining and Technology, Xuzhou 221116, China; 17769685084@163.com (H.M.); zengwentian023@163.com (W.Z.); 3 State Key Laboratory of Environmental Adaptability for Industrial Products, China National Electric Apparatus Research Institute Co., Ltd., Guangzhou 510663, China; xuk@cei1958.com; 4 State Key Laboratory of Power Transmission Equipment Technology, School of Electrical Engineering, Chongqing University, Chongqing 400044, China; zhangzhijing@cqu.edu.cn

**Keywords:** damp-heat ageing, epoxy resin, phenolic resin, flame retardancy, oxygen index, vertical combustion test, scanning electron microscopy (SEM), energy-dispersive spectroscopy (EDS), insulation materials

## Abstract

As power equipment insulation materials, epoxy resin and phenolic resin are inevitably exposed to damp-heat environments during long-term operation, leading to ageing degradation. This study systematically investigates the effects of damp-heat ageing (85 °C, 90% RH, 0–56 days) on the flame retardancy of both resins through oxygen index tests, vertical combustion experiments, and microstructural analysis (SEM/EDS). Results indicate that ageing unexpectedly enhances flame retardancy: Epoxy resin: Oxygen index rapidly increased from 64.4% (0 days) to 76.5% (21 days), then stabilised at 75–76%. Afterflame time decreased from 143 s to 109 s after 56 days. Phenolic resin: Oxygen index rose continuously with ageing; afterflame time dropped sharply from 211 s (0 days) to 0 s (56 days). Mechanistic analysis reveals that ageing promotes surface enrichment of flame-retardant elements (Ca, Mg, Si in epoxy; Al, Mg in phenolic) and structural changes (e.g., porous carbonisation), which facilitate barrier effects against heat/oxygen diffusion. This work challenges conventional views on ageing-induced degradation, providing new insights for evaluating insulation safety in humid environments.

## 1. Introduction

Resin materials are currently widely used in power systems and are an important classification of insulation materials [1,2,3,4]. However, a large part of the insulation materials used in power equipment are organic materials that are flammable [5,6]. Flame retardants are usually added to insulation materials to prevent ignition and suppress flame propagation. However, power equipment is subjected to various stresses during operation, such as electrical stress, thermal stress, etc. Under these stresses, insulation materials will continuously age with increasing operating time [7,8,9,10,11], causing a decrease in insulation and flame-retardant properties, greatly increasing the risk of fire in power equipment.

Epoxy resin and phenolic resin are the two most commonly used types of resin materials in power systems. Taking epoxy resin as an example, it is currently the most widely used thermosetting polymer material in the field of engineering materials [12,13,14]. Due to its excellent adhesion, heat resistance, mechanical properties, electrical insulation properties, etc., it is widely used in coatings, adhesives, composite materials, castables and other fields. In the power system, epoxy resin is widely used in power equipment such as dry-type transformers, dry-type reactors, bushings, and supporting insulators due to its excellent comprehensive performance and designability.

In practical applications, epoxy resin in power equipment undergoes ageing due to long-term exposure to strong electric fields, high temperatures, and environmental factors [15,16,17,18]. Epoxy resin may experience a decrease in insulation performance, increased local losses, abnormal overall heating, inter-turn short circuits, local overheating, and other forms of faults, which can lead to accidents such as fire and burnout. In order to ensure the safe and stable operation of power equipment using epoxy resin throughout its entire life cycle, it is necessary to evaluate and predict the flame-retardant performance of epoxy resin after multi-factor ageing.

A large amount of studies have been conducted on the ageing of resin materials [19,20,21,22,23]. One such study [19] examines the corona ageing characteristics of epoxy resin under different hygrothermal environments. Research has found that higher environmental temperatures or relative humidity can exacerbate the internal degradation of aged epoxy resin samples, making polar groups more prone to rotation, resulting in polarisation relaxation loss and dielectric loss. The research results aim to provide reference for the operation and maintenance of different electrical equipment using epoxy resin materials in harsh environments. Another study [21] covered the effect of thermal oxidation ageing at different times on the dielectric properties of epoxy resin. Research has found that the competitive results of molecular rearrangement, post-curing, and oxidative cracking reactions during thermal ageing determine the insulation performance of epoxy resins, providing a theoretical basis for the ageing evaluation of high-voltage epoxy resins.

However, there is limited research on the impact of ageing on flame retardancy of resin materials. Only a small amount of literature has been used to study the effect of flame-retardant addition on ageing characteristics [24,25]. For example, reference [24] studies the thermo-oxidative ageing of epoxy resin with decabromodiphenylethane (DBDPE). According to the results, it is concluded that DBDPE in EP can accelerate thermo-oxidative ageing. These results can provide theoretical guidance for the engineering application of DBDPE-filled EP.

However, the impact of ageing on the flame retardancy of resin insulation materials used in power equipment remains largely unexplored. Therefore, by simulating actual ageing conditions and combining combustion performance tests with microscopic characterisation, we aim to reveal the evolution laws of flame retardancy for epoxy resin and phenolic resin during hygrothermal ageing, providing theoretical support for the life assessment and safety design of insulation materials.

## 2. Experimental Methods

### 2.1. Samples and Devices

The samples consisted of epoxy resin and phenolic resin materials used as insulation components for power equipment. The dimensions of the strip specimens were length = 125 ± 5 mm, width = 13.0 ± 0.5 mm, and thickness = 3.0 ± 0.1 mm. A total of 70 strip specimens were prepared, divided into seven groups (S0~S6), with 10 specimens per group.

**Figure 1:**
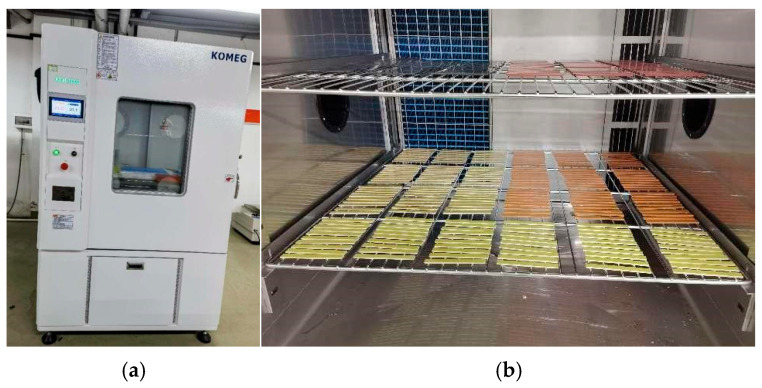
Wet heat ageing test equipment and test process. (**a**) Test chamber. (**b**) Sample layout diagram.

A KFH-1000SF1A0 high- and low-temperature humidity test chamber was used for the samples’ humidity and heat ageing tests. The test device and samples are shown in Figure 1.

The oxygen index was tested using an oxygen index meter. A HVUL-2 (AMETEK, West Chicago, IL, USA) combustion instrument, with CH4 as the combustion gas, was used for a vertical combustion test. Scanning electron microscopy (SEM, MIRA LMS, Brno, Czech Republic.) and energy-dispersive spectroscopy (EDS, XFlash 6160, Bruker in Berlin, Germany) were used to test the sample’s surface microstructure and elemental distribution. To prevent potential deviations in experimental results caused by surface oxidation or changes in cross-linking density during long-term static storage at room temperature, the samples were subjected to SEM, EDS, and combustion performance testing immediately following the ageing treatment. This procedure ensures that the characterisation captures the material’s condition as it would appear under real-world energised operation.

The epoxy and phenolic resins utilised in this study were sourced directly from materials used in actual manufacturing equipment. Due to proprietary concerns regarding formulation confidentiality, detailed compositional data were not provided by the manufacturer. To address this limitation, energy-dispersive spectroscopy (EDS) was performed on unaged samples to determine their elemental content, which serves as a reference for the initial composition.

### 2.2. Wet Heat Ageing Test

According to the IEC 60068-2-78:2012 standard [26], the samples underwent a wet heat ageing test. The ambient temperature was 85 ± 5 °C, and the relative humidity was 90 ± 10%. The experiment consisted of one control group (S0) and six ageing groups (S1–S6). S0 represented the unaged samples, while S1 to S6 corresponded to ageing durations of 1, 4, 10, 21, 35, and 56 days, respectively.

During the test, the initial temperature was 20 °C, which was raised to 85 °C within 2 h. The humidity parameters were set through the electronic control screen. Through the gradient setting, the humidity of the experimental environment was maintained at around 90%. Based on the relevant experimental standards, the corresponding ageing time was maintained according to the current wet and hot environments. Then, the experimental device was closed to complete the experimental process. After the damp and hot ageing test, the samples were pre-treated at 23 ± 2 °C and a relative humidity of 50 ± 5% for at least 48 h.

### 2.3. Test Method for the Flame-Retardant Properties of Resin Sample

Vertical combustion test: According to the IEC 60695-11-10 standard [27], a vertical combustion test was carried out on the relevant test samples. The vertical burning test was designed to measure the self-extinguishing ability of a material under specified test conditions. One end of the strip sample was held horizontally during the test, and the free end was in contact with the specified test flame. The flame height was 20 mm. The first flame was applied for 10 s, and the afterflame time t1 was recorded. The second flame was applied for 10 s, and the afterflame time t2 and afterburning time t3 were recorded or applied until the flame reached the 25 mm mark. The burning time between the 25 and 100 mm marks was recorded. Upon completion of the test, a repeatability test was conducted to verify the results. The verified results showed a small deviation from the initial trial and met the standard requirements; therefore, the obtained results are considered reliable.

Ultimate oxygen index determination test: The oxygen index refers to the minimum oxygen concentration required to maintain the combustion of a material when a mixture of oxygen and nitrogen at a temperature of 23 ± 2 °C is introduced under specific conditions. During the measurement, the sample was fixed vertically in a transparent combustion tube filled with an upward-flowing mixture of oxygen and nitrogen. The top of the sample was ignited, and a series of tests under different oxygen concentrations were conducted by comparing the continuous combustion time or combustion length of the sample to estimate the minimum oxygen concentration required to maintain combustion.

The surfaces of the samples were observed using scanning electron microscopy, with five keV employed for examining the samples. Furthermore, the distribution of the elements was obtained by energy-dispersive spectroscopy. Before testing, the sample was cleaned using an ultrasonic cleaning machine to remove surface dust. Next, a gold sprayer (CCU-010) was used to spray gold to ensure the conductivity of the sample so that SEM and EDS testing could be performed.

## 3. Effect of Wet Heat Ageing on the Combustion Characteristics of Resin Sample

### 3.1. Vertical Burning Phenomenon During the Wet Heat Ageing Process of Epoxy Resin

**Figure 2:**
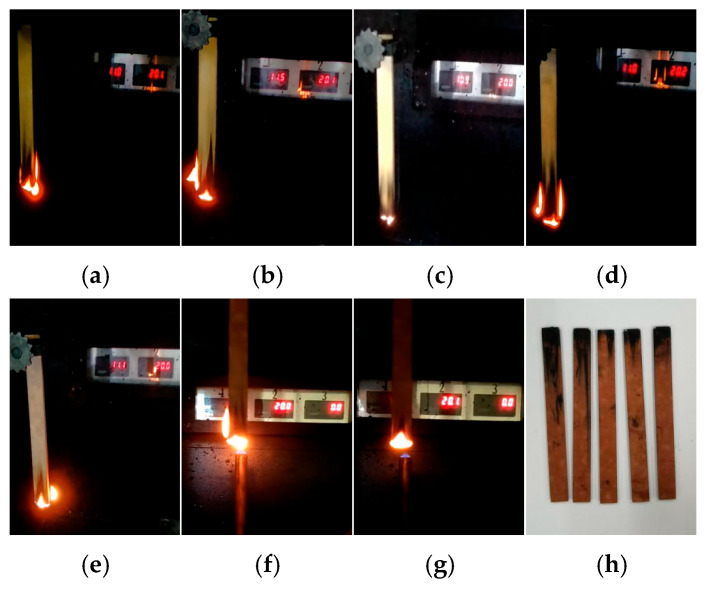
Vertical combustion phenomenon of epoxy resin at different ageing times. (**a**) S0-30 s. (**b**) S1-30 s. (**c**) S2-30 s. (**d**) S3-30 s. (**e**) S4-30 s. (**f**) S5-30 s. (**g**) S6-30 s. (**h**) S6.

Taking the 30 s vertical combustion test as an example, the combustion phenomena of epoxy resin samples with different ageing days are shown in Figure 2.

From the figure, it can be seen that firstly, the colour of the sample changes, and with the increase in ageing time, the colour of the epoxy resin gradually deepens. This is usually because at high temperatures, the molecular chains of epoxy resin break or rearrange, forming conjugated double bonds (-C=C-C=C-). The longer the conjugated system, the longer the absorption wavelength of visible light, and the colour gradually deepens (such as from yellow to brown).

**Figure 3:**
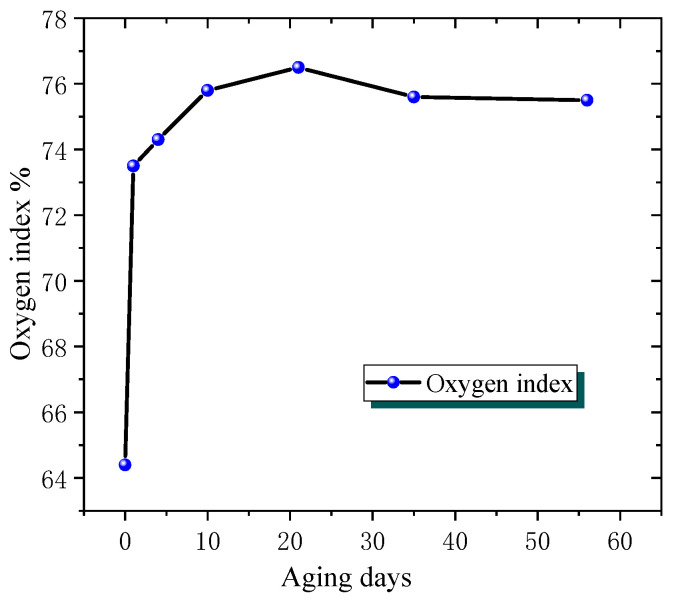
Oxygen index change of epoxy resin with ageing time.

**Figure 4:**
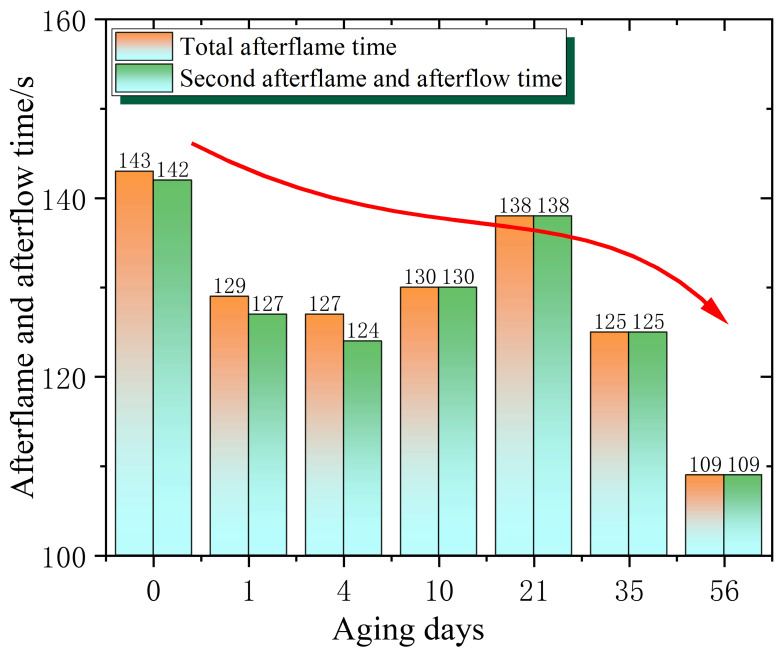
Epoxy resin afterflame and afterglow time changes with ageing time.

In addition, it can be observed from the combustion phenomenon that, interestingly, the flame retardancy appears to improve in the late stage of ageing. To characterise the flame retardancy in more detail, the afterflame and afterflow times of combustion, as well as the oxygen index of the sample, were further obtained, as shown in Figure 3 and Figure 4.

As shown in Figure 3, during the initial stage (approximately 0–21 days), the oxygen index exhibits a rapid upward trend, increasing from approximately 64.4% to nearly 76.5%. This may be because in the early stages of ageing, epoxy resin undergoes some changes that are beneficial for improving flame retardancy, such as the formation of more conjugated structures, nitrogen/phosphorus-containing flame-retardant groups, or an increase in cross-linking degree, making the material more difficult to ignite during combustion and increasing the oxygen index.

However, after 21 days, the oxygen index reached a relative peak and began to decrease slightly, but the decrease was small, remaining between 75% and 76%. At this point, it may be due to the gradual balance of certain factors that promote flame retardancy during the ageing process, or subtle changes occurring within the material, such as slight damage to carbon layer structures, while overall maintaining high flame retardancy.

From Figure 4, the same conclusion as Figure 3 is obtained. With the increase in ageing time, the overall flame retardancy of epoxy resin shows an upward trend. For example, in the initial stage (0 days), the total afterflame time is 143 s, and the second afterflame and afterflow times are 142 s, with higher values indicating that the epoxy resin continues to burn and emit light for a longer time after combustion in the initial state. However, after 56 days of ageing, it decreased to 109 s. As the ageing time is further prolonged, the flame-retardant performance of the material continues to improve, and the duration of continuous combustion and luminescence after combustion is continuously shortened.

### 3.2. Vertical Burning Phenomenon During the Wet Heat Ageing Process of Phenolic Resin

Taking the 21 s vertical combustion test as an example, the combustion phenomena of phenolic resin samples with different ageing days are shown in Figure 3.

**Figure 5:**
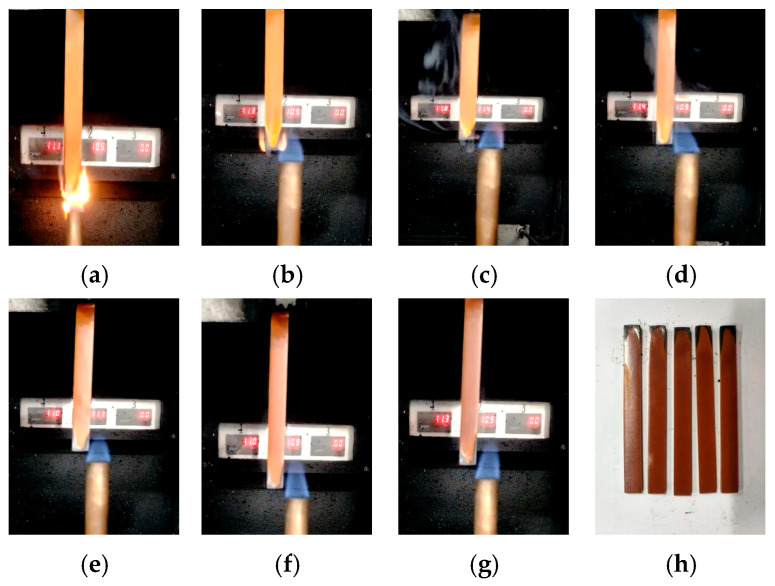
Vertical combustion phenomenon of phenolic resin at different ageing times. (**a**) S0-21 s. (**b**) S1-21 s. (**c**) S2-21 s. (**d**) S3-21 s. (**e**) S4-21 s. (**f**) S5-21 s. (**g**) S6-21 s. (**h**) S6.

**Figure 6:**
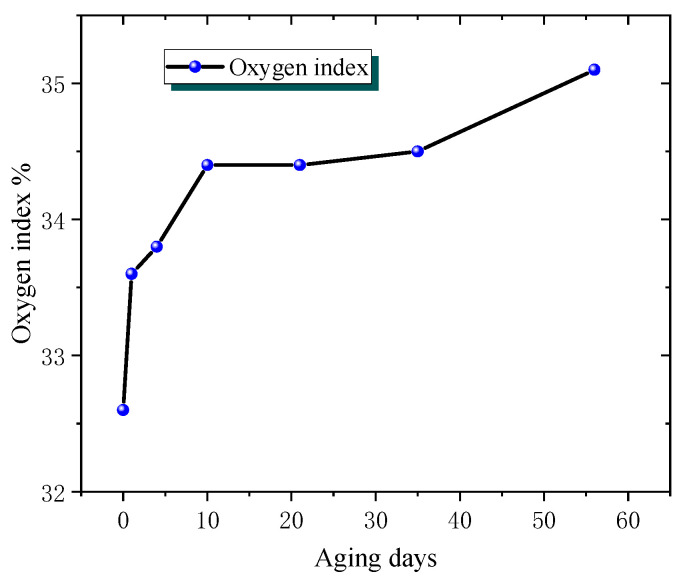
Oxygen index change of phenolic resin with ageing time.

**Figure 7:**
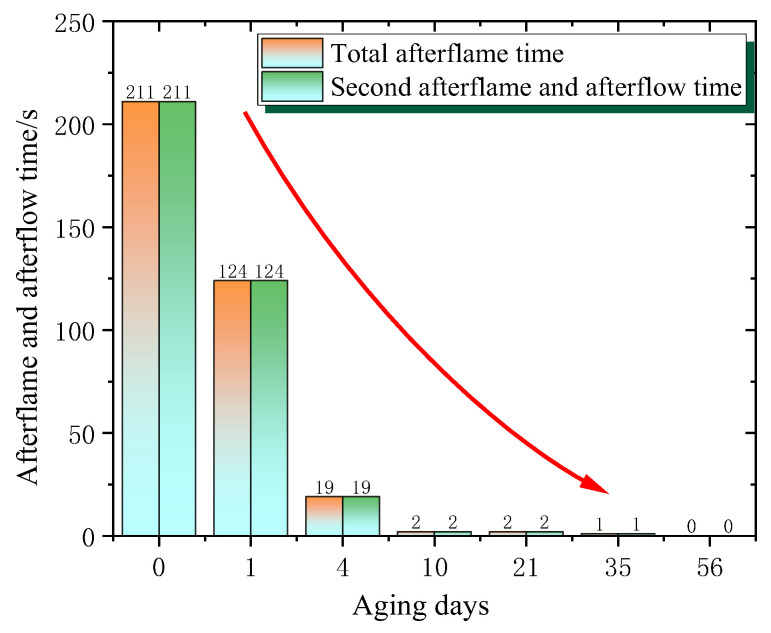
Phenolic resin afterflame and afterglow time changes with ageing time.

From Figure 5, it can be intuitively seen that the flame-retardant performance of phenolic resin after ageing is similar to that of epoxy resin. The flame retardancy appears to improve in the late stage of ageing. Unlike epoxy resin, the effect of ageing on the flame retardancy of phenolic resin seems to be more pronounced. To characterise the flame retardancy in more detail, the afterflame and afterflow times of combustion, as well as the oxygen index of the sample, were further obtained, as shown in Figure 6 and Figure 7.

Figure 6 clearly shows the dynamic changes in the oxygen index of phenolic resin during the ageing process, which first increases rapidly, then transitions smoothly, and finally increases again, intuitively reflecting the impact of the ageing process on the flame-retardant properties of phenolic resin.

Comparing the two resin materials, there is a rapid increase in oxygen index during the early stages of ageing, but epoxy resin shows a brief decrease and then stabilises, while phenolic resin continues to rise after a smooth transition. This reflects the differences in the internal chemical structure and reaction process of the two resins during ageing, resulting in different evolution patterns of flame-retardant properties with ageing time.

As shown in Figure 7, during the initial stage (0 days), both the total afterflame time and the second afterflame and afterflow time of phenolic resin are 211 s. The initial values are higher than those of epoxy resin, indicating that the phenolic resin has an even longer duration of continuous combustion and luminescence after burning in the initial state.

Sharp decline stage (about 0–4 days): When aged for 1 day, the total afterflame time and the second afterflame and afterflow time drop to 124 s; when aged for 4 days, they drop sharply to 19 s. This indicates that during the initial ageing stage, drastic changes occur within the phenolic resin, rapidly enhancing its flame-retardant performance and significantly reducing the duration of continuous combustion and luminescence after burning.

Extremely short and stable stage (about 4–56 days): From 4 days to 56 days of ageing, the afterflame and afterflow times remain at an extremely low level. For example, they are both 2 s when aged for 10 days and 21 days, 1 s when aged for 35 days, and 0 s when aged for 56 days. This indicates that after a short period of ageing, the flame-retardant performance of the phenolic resin reaches a very high level, and it hardly continues to burn and luminesce after burning.

It is worth noting that this study employed the Limiting Oxygen Index and vertical burning tests for a preliminary assessment of the samples’ combustion performance. To further investigate properties such as released components and heat generation during combustion, additional tests including cone calorimetry are required to obtain a more comprehensive understanding of the evolution of combustion behaviours during material ageing.

### 3.3. Analysis of the Impact of Ageing on Flame Retardancy

**Figure 8:**
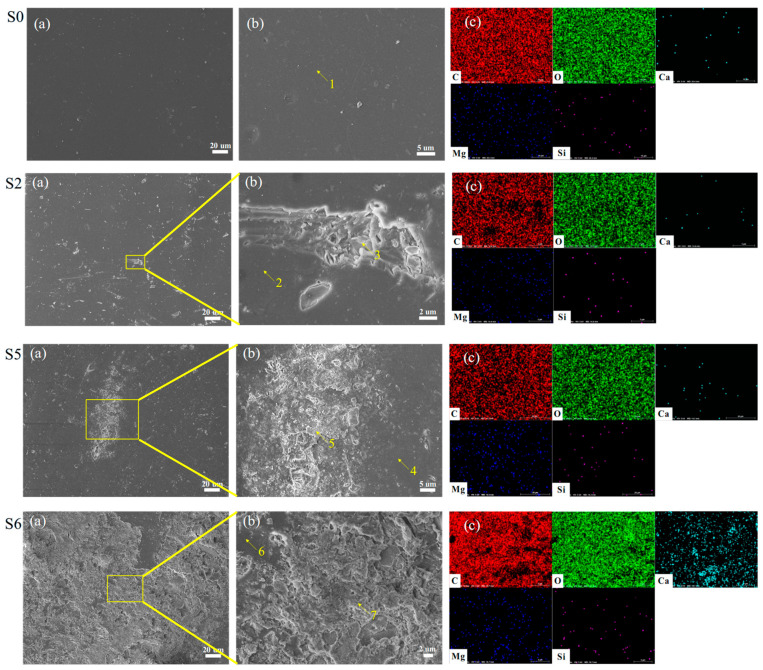
Surface observation morphology and elemental analysis of epoxy resin. (**a**) SEM image at 1000× magnification. (**b**) Locally magnified SEM image of the characteristic point. (**c**) Elemental distribution map of the local characteristic point.

**Figure 9:**
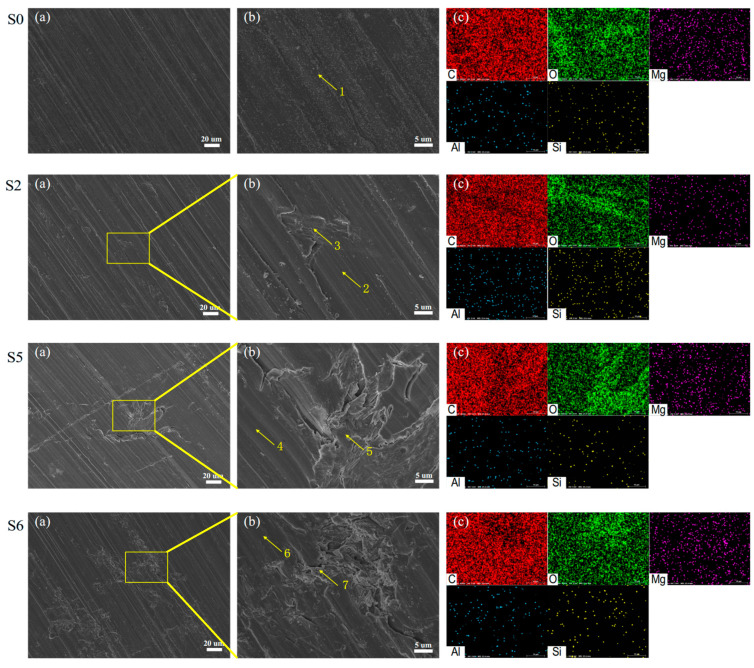
Surface observation morphology and elemental analysis of phenolic resin. (**a**) SEM image at 1000× magnification. (**b**) Locally magnified SEM image of the characteristic point. (**c**) Elemental distribution map of the local characteristic point.

To analyse the reasons for the influence of ageing on flame retardancy, the surface microscopic morphology and element distribution of epoxy resin and phenolic resin with different degrees of ageing were characterised, and the results are shown in Figure 8 and Figure 9. Scanning electron microscopy images illustrate the changes in the surface micro-morphology of the hygrothermal ageing samples. Due to the dispersion of microscopic features, only a qualitative analysis is conducted here to compare the differences in surface morphology among samples with varying degrees of ageing.

The energy spectrum analysis results indicate that the epoxy resin used in this study is primarily composed of C, O, Ca, Mg, and Si, while the phenolic resin mainly consists of C, O, Mg, Al, and Si. Among these, carbon and oxygen constitute the main backbone of both materials, whereas the other elements originate from additives introduced to enhance properties such as mechanical performance.

From Figure 8, it can be seen that the S0 sample, as the initial state, has a relatively flat and smooth surface, indicating that the epoxy resin structure is uniform before ageing.

After 4 days of ageing, some subtle changes begin to appear on the surface, such as small protrusions or irregular structures in local areas (arrow 3), indicating that ageing is beginning to affect the surface structure.

After 35 days of ageing, the surface changes become more pronounced, with block or sheet-like structures appearing (arrow 5), surface roughness increasing, and the material structure undergoing significant changes due to ageing.

After 56 days of ageing, the surface showed complex porous or grooved structures (arrow 7), indicating that ageing continued to damage the structure of the epoxy resin, making its surface more porous and defective.

The loosened structure induced by ageing may increase the specific surface area of the material, thereby facilitating the dispersion and efficacy of flame retardants. Meanwhile, a high specific surface area could accelerate the carbonisation reaction during combustion. The resulting char layer covering the material surface further prevents heat transfer, insulates oxygen, and hinders the escape of combustible gases, thus suppressing the continuation of combustion.

**Figure 10:**
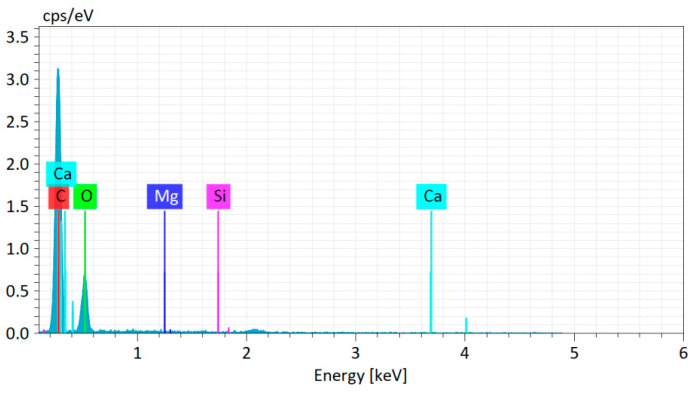
Element content test results.

The morphology of phenolic resin after ageing, shown in Figure 9, has similar characteristics to those in Figure 8. With increasing ageing time, there is obvious structural damage and precipitation of certain substances on the surface. To further analyse the composition of the precipitated substances on the surface, quantitative analysis was conducted on the elements at the points marked in Figure 8 and Figure 9. The test results of one point are shown in Figure 10. The results of counting all points are shown in Table 1 and Table 2.

Generally, as hygrothermal ageing progresses, the C content of the epoxy resin exhibits an overall decreasing trend, while O content increases. This is primarily attributed to the scission of benzene rings at elevated temperatures and the intrusion of hydroxyl groups -OH from water molecules into the cleavage sites, resulting in decreased surface C and increased O. The rupture of the carbon skeleton induces microscopic local stress inhomogeneity within the epoxy resin, further leading to macroscopic features such as surface cracking, a phenomenon consistent with the observations in Figure 8. Meanwhile, in the later stages of hygrothermal ageing, the levels of Ca, Mg, and Si on the sample surface rise significantly. Ca typically exists as CaCO_3_ acting as a reinforcing agent, Mg as Mg(OH)_2_ serving as a flame retardant, and Si as SiO_2_ functioning as another reinforcing agent. The increase in these three elements indicates that additives accumulate on the surface during hygrothermal ageing. This accumulation not only alters the flame retardancy of the epoxy resin but also indirectly contributes to the observed rise in surface O content due to the enrichment of oxygen-containing additives.

From Table 1, it can be seen that for the C element, point 1 is the non-aged position, with a C element content of up to 77.14 wt%, which is the main constituent element of epoxy resin. When not aged, the content is at a high level, representing the initial molecular skeleton structure state of epoxy resin.

For feature points 3, 5, and 7 with surface defects and other morphologies, point 3 has a C element content of 88.49 wt%, which is higher than point 1. This may be due to the loss of some other elements or chemical reactions during the ageing process, increasing the relative proportion of the C element. The C element content at point 5 has significantly decreased to 30.07 wt%, indicating severe damage to the molecular structure caused by ageing. A large number of C-C bonds have been broken, resulting in a sharp decrease in the C element content. The content of C element in point 7 is 18.01 wt%, further indicating that ageing causes severe degradation of the molecular structure of epoxy resin and significant loss of C element.

The increase in the O element indicates that an oxidation reaction occurs during the ageing process, introducing a large number of oxygen atoms, which may form new oxygen-containing functional groups. The content of the O element in point 7 is 53.95 wt%, further proving that the degree of oxidation deepens in the later stage of ageing.

Furthermore, interestingly, an increase in and enrichment of surface Ca and Mg elements were discovered. Taking Ca as an example, the Ca content in point 1 is only 0.54 wt%, the Ca content in point 3 is 1.33 wt%, the Ca content in point 5 is as high as 10.47 wt%, and the Ca content in point 7 is 8.38 wt%. During the ageing process, the content of the Ca element significantly increases, which may be due to the loosening of the material structure caused by ageing. The originally trace calcium-containing components on the surface migrate and accumulate from the inside to the surface during the ageing process. In addition, as the Si element content increases with ageing, it may be due to the interaction between the material and silicon-containing substances during the ageing process, such as adsorption of silicon-containing impurities, reaction with silicon-containing additives, etc.

According to the results of elemental analysis, the improved flame retardancy after ageing may be attributed to the following reasons.

The increase in Ca and Mg elements indicates that fillers such as calcium compounds (such as calcium carbonate) and magnesium compounds (such as magnesium hydroxide) in the material gradually precipitate with the increase in ageing. Calcium carbonate can decompose and absorb heat at high temperatures, reducing the surface temperature of the material and inhibiting combustion reactions. The gases, such as carbon dioxide, produced by simultaneous decomposition can dilute the concentration of combustible gases, block oxygen, and have a flame-retardant effect. Magnesium hydroxide is a common flame retardant that absorbs a large amount of heat during thermal decomposition, reducing the material temperature; the water vapour generated by decomposition can dilute the concentration of combustible gases and oxygen; and the decomposed magnesium oxide covers the surface of the material, forming a thermal and oxygen barrier to prevent combustion from continuing.

After ageing, the content of the Si element increases, which may be due to the interaction between the material and silicon-containing substances. Silicon-containing compounds (such as organosilicon resins) can form a protective film with siloxane structure on the surface of materials, which has good thermal and oxygen insulation properties and can suppress combustion; it may also promote the formation of a carbon layer, improve its strength and stability, and enhance its flame-retardant effect.

The significant increase in O element content at ageing sites 5 and 7 indicates the formation of new oxygen-containing functional groups through oxidation reactions during the ageing process. Partial oxygen-containing structures can promote material dehydration and carbonisation at high temperatures, forming a carbon layer that prevents heat transfer and isolates oxygen, thereby enhancing flame retardancy.

The change in the C element indicates that ageing causes severe degradation of the molecular structure of epoxy resin. The degradation process may produce small molecular fragments with flame-retardant properties, or promote the formation of a carbon layer in the material, which can play a role in insulation, oxygen isolation, and preventing the escape of flammable gases, thereby improving flame-retardant performance.

As hygrothermal ageing progresses, the phenolic resin also exhibits a decrease in C content and an increase in O content, accompanied by the surface enrichment of internal additive elements. However, compared to epoxy resin, the variation in elemental content is slightly smaller. This indicates that hygrothermal ageing involves the scission of benzene rings and methyl groups, as well as an increase in hydroxyl groups -OH. The surface enrichment of additives leads to increased levels of Si, Mg, and Al on the surface, which significantly affects the combustion performance of the phenolic resin, resulting in enhanced flame retardancy after ageing.

Compared with epoxy resin, the element distribution characteristics of phenolic resin are different. For example, the changes in O and C elements on the surface of phenolic resin after ageing are relatively weak. This indicates that ageing did not significantly degrade the molecular structure of phenolic resin, which can also be confirmed from the microstructure. But the similarity is that the Mg element increases with ageing. In addition, an increase in the Al element was found on the surface of the phenolic resin. The increase in Al and Mg elements indicates that fillers such as aluminium-containing compounds (such as aluminium hydroxide) and magnesium-containing compounds (such as magnesium hydroxide) in the material gradually precipitate with increasing ageing. For example, aluminium-containing compounds (such as aluminium hydroxide) absorb heat through thermal decomposition, lowering the material temperature. The water vapour generated by decomposition dilutes the concentration of combustible gases and oxygen. At the same time, the aluminium oxide generated by decomposition can form a protective film on the surface of the material, blocking heat and oxygen, and playing a flame-retardant role.

For phenolic resin, on the one hand, ageing does not significantly degrade the molecular structure of phenolic resin, and on the other hand, ageing causes flame retardants to accumulate on the surface. Therefore, it greatly increases the flame-retardant performance of the aged material, which is different from conventional understanding. This research result will provide a new approach and reference for evaluating the flame retardancy of aged materials.

A comparison of the hygrothermal ageing behaviours between epoxy resin and phenolic resin reveals that while both materials share numerous similar ageing characteristics, distinct differences also exist. The ageing process of epoxy resin is more susceptible to external environmental influences, leading to significant performance variations. This is manifested in elemental changes as extensive scission of benzene rings causing a sharp rise in oxidation, which results in a substantial decrease in C content and a marked increase in O content [28]. As the carbon skeleton reconstructs, internal additives accumulate on the surface to a greater extent, significantly affecting combustion performance; this is reflected in the flammability tests as a substantial increase in the Limiting Oxygen Index of the epoxy resin.

## 4. Conclusions

This study demonstrates that damp-heat ageing (85 °C, 90% RH) significantly enhances the flame retardancy of epoxy and phenolic resins, contrary to conventional degradation expectations:

(1) Epoxy resin: Oxygen index increased by 19% (64.4%→76.5%) within 21 days, while afterflame time decreased by 24% (143 s→109 s) after 56 days. Ageing induces surface enrichment of Ca/Mg/Si (e.g., Ca content surged from 0.54 wt% to 10.47 wt%) and forms porous carbonised structures, promoting heat absorption and oxygen barrier effects.
(2) Phenolic resin: Achieved near-complete self-extinguishing (afterflame time: 211 s→0 s) after 56 days. Flame-retardant fillers (Al/Mg compounds) migrate to the surface without significant molecular degradation, enhancing dehydration and carbon layer formation.
(3) Mechanistic insights: Ageing facilitates the precipitation of inherent flame-retardant additives (e.g., CaCO_3_, Mg(OH)_2_, Al(OH)_3_) and generates oxygen-containing functional groups, accelerating protective carbon layer formation. Structural loosening increases the specific surface area, thereby improving the dispersion efficiency of flame-retardant elements.
(4) Engineering Implications: The counterintuitive changes in the flame retardancy of epoxy resin and phenolic resin under laboratory hygrothermal ageing suggest that aged insulation materials may possess higher safety levels than previously assumed. However, the specific variation patterns require future verification using field samples. These findings can be further utilised to optimise resin formulations to harness ageing-induced flame-retardant mechanisms.

## Figures and Tables

**Table 1:** Quantitative results of element distribution at different points on the surface of epoxy resin.

Pionts	Element (wt%)				
	C	O	Ca	Mg	Si
1	77.14	22.32	0.54	0.00	0.00
2	75.75	22.77	0.69	0.40	0.39
3	88.49	15.09	1.33	4.24	0.85
4	71.77	26.55	0.82	0.39	0.47
5	30.07	51.65	10.47	6.71	1.10
6	68.29	29.92	0.94	0.31	0.54
7	18.01	53.95	8.38	14.37	5.29

**Table 2:** Quantitative results of element distribution at different points on the surface of phenolic resin.

Pionts	Element (wt%)				
	C	O	Si	Mg	Al
1	70.41	28.47	0.70	0.32	0.10
2	80.65	19.07	0.24	0.00	0.04
3	78.47	20.77	0.41	0.21	0.14
4	72.12	27.33	0.53	0.02	0.00
5	73.61	26.13	0.73	0.26	0.10
6	76.76	23.07	0.17	0.00	0.00
7	60.90	34.41	2.55	1.28	0.86

## Data Availability

The raw data supporting the conclusions of this article will be made available by the authors on request.
